# Incidental Finding of an Exceptionally Large Left Atrial Myxoma Presenting as an Acute Cardioembolic Stroke

**DOI:** 10.7759/cureus.18056

**Published:** 2021-09-17

**Authors:** Rebecca A Bedoya, Trevor Smith, Hoan Ma, Amy Goodner, Jason Sreedhar

**Affiliations:** 1 Family Medicine, Broward Health Medical Center, Fort Lauderdale, USA; 2 Family Medicine, Nova Southeastern University Dr. Kiran C. Patel College of Osteopathic Medicine, Clearwater, USA; 3 Family Medicine, Nova Southeastern University Dr. Kiran C. Patel College of Osteopathic Medicine, Fort Lauderdale, USA

**Keywords:** family medicine, cardiothoracic surgery, echocardiogram, stroke, mca occlusion, tumor, left atrial myxoma

## Abstract

Left atrial myxomas are rare tumors that arise in the left atrium of the heart. As they become larger, they tend to grow into the atrial lumen and disrupt cardiac hemodynamics. Commonly reported symptoms include dyspnea, orthopnea, cough, peripheral edema, and fatigue. On physical examination, a characteristic “tumor plop” may be heard in some patients early in diastole. Left atrial myxomas may cause emboli to be released into the systemic circulation, which can lead to acute cardiovascular events, including strokes. We present the case of a 43-year-old female with sudden-onset slurred speech, left facial droop, and left-sided hemiplegia. CT angiography of the brain revealed a right middle cerebral artery infarct, and the patient underwent emergent mechanical thrombectomy. Upon workup for secondary causes of stroke, echocardiogram revealed an incidental 8 cm left atrial myxoma. After stabilization in the ICU, the patient was taken to surgery and the tumor was successfully removed. Over the course of admission, the patient’s left-sided hemiplegia gradually improved, and she was eventually transferred to inpatient rehabilitation care. A multidisciplinary effort involving medicine teams, neurology, cardiology, cardiothoracic surgery, neuro-interventional radiology, pain management, and endocrinology was essential in reaching the diagnosis. This case highlights the importance of considering a primary cardiac tumor such as a left atrial myxoma in the differential diagnosis when evaluating for secondary causes of acute ischemic stroke.

## Introduction

Primary cardiac tumors are rare with an incidence as low as 0.03%, and 75% of which are benign. Myxomas make up 50% of those benign cases. Left atrial myxomas are relatively benign primary cardiac tumors with an incidence rate of 0.5% per million per year [1]. The peak incidence of a cardiac myxoma occurs in 50-60-year-old females [2]. Proposed etiologies for cardiac myxomas include entrapment of embryonic foregut within the cardiac tissue, allowing for neural and epithelial differentiation [3].

Myxomatous tumors of the left atrium (LA) induce pathologic symptoms through a myriad of mechanisms. By obstructing flow through heart chambers and/or valves, LA myxomas can cause heart failure or mitral regurgitation [4]. Myxomas can invade the local cardiac or pulmonary tissue, causing hypo-contractility, arrhythmia, pericardial effusion, cardiac tamponade, or invasion of the lung tissue [4, 5]. Finally, the release of fragments or thrombi from the tumor can cause embolic pathologies, the most devastating of which are neurologic [5, 6]. The tumors themselves will most commonly present as 1-15 cm masses ranging from 15 to 180 g and grow at a rate of 1.3-6.9 mm/month [5, 7]. Larger tumors will present with a smoother surface, while smaller tumors are generally friable or villous in appearance and thus are more susceptible to embolization [5]. Histologically, myxomas demonstrate scattered and unstructured cells on a myxoid stromal background of mucopolysaccharides and glycosaminoglycans [5, 8].

Atrial myxomas classically present with non-specific general and cardiorespiratory symptoms, most commonly: dyspnea; orthopnea; paroxysmal nocturnal dyspnea; pulmonary edema; cough; hemoptysis; peripheral edema; and fatigue [4]. Fever and weight loss are seen in 30% of patients, while laboratory abnormalities (anemia, increased globulins, increased erythrocyte sedimentation rate (ESR), increased C-reactive protein (CRP)) are seen in 35% of patients [5, 9]. On a physical exam, a “tumor plop”-a low-pitched diastolic sound immediately after S2-can be appreciated based on the position of the patient [5, 10].

Echocardiogram represents a simple and non-invasive diagnostic avenue, providing pictorial evidence of the tumor mass, circulation obstruction, and sources of emboli [5, 10]. While transthoracic echocardiograms are less invasive, transesophageal echocardiograms provide improved imaging due to improved spatial resolution sensors and less obstructing tissue [4, 5]. Other diagnostic methodologies include cardiac MRI, cardiac CT, and PET scans (differentiate myxoma vs metastasis to heart) [4, 5]. Transvenous biopsies are not recommended due to the risk of embolism [4, 5, 6].

Treatment of LA myxomas focuses on prompt surgical resection to minimize the possibility of embolic or cardiac complications [5, 6]. Resection is classically done through median sternotomies, but newer alternatives including right anterolateral mini thoracotomies entail a smaller risk of infection or cosmetic issues [6]. Post-surgical prognosis is generally good (<5% intraoperative mortality rate) with rapid recovery [4, 5]. Post-surgical complications include arrhythmias, nodal conduction abnormalities, infection, and tumor recurrence (5% of cases) [4, 5]. Cardiac transplant is indicated for recurrent LA myxomas [5].

## Case presentation

A 43-year-old female with a past medical history of hypothyroidism presented to the emergency department with slurred speech, left facial droop, and left-sided hemiplegia. At the time of presentation, she denied any chest pain, shortness of breath, fever, chills, nausea, and vomiting. Patient reported a recent history of mild chest pain and shortness of breath when walking. She had a cardiologist but has not had an echocardiogram or stress test performed. Patient did not have any significant past surgical history or family history of strokes, hypertension, or diabetes mellitus. The only medication the patient was taking was levothyroxine 300 mg daily. She denied alcohol use but endorsed a 28-pack-year smoking history.

Upon examination, the patient was afebrile with a blood pressure of 129/80 mmHg, heart rate of 79 beats/min, respiratory rate of 17/min, and oxygen saturation of 98% on room air. She had a BMI of 43.03. On a cardiovascular exam, the patient had a normal rate and rhythm, S1 and S2, no murmurs noted. The neurologic exam revealed tongue deviation to the left, 5/5 strength in the right upper and lower extremity with intact sensation, 0/5 strength in the left upper and lower extremity with impaired sensation.

**Figure 1:**
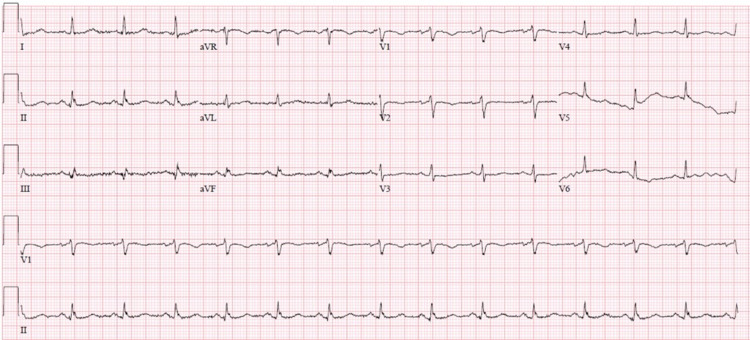
EKG showing normal sinus rhythm, low voltage QRS, and prolonged QT interval

Admission laboratory studies revealed no acute abnormalities except for an elevated thyroid-stimulating hormone (TSH) at 15.15 (normal <4.94) with an elevated thyroid peroxidase antibody of 99 (normal <9), leading to a clinical diagnosis of Hashimoto Thyroiditis. EKG on admission (Figure 1) revealed normal sinus rhythm with a rate of 84 beats/minutes, low voltage QRS, and a prolonged QT interval.

**Figure 2:**
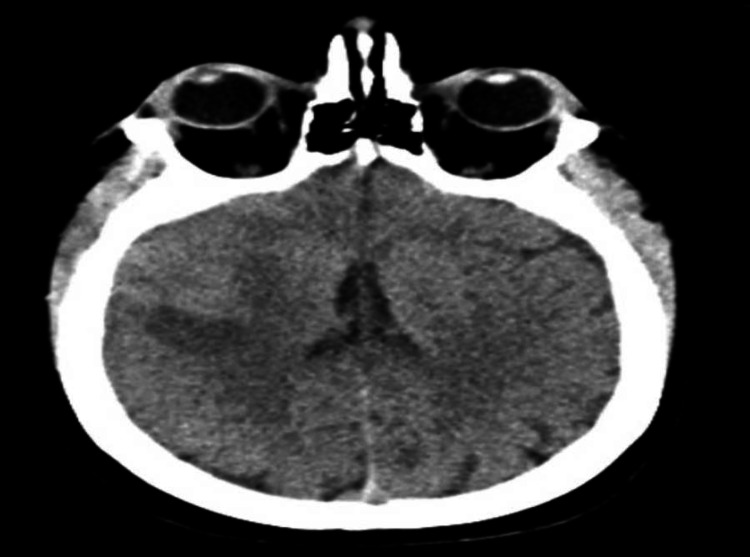
Hypoattenuating area within the right MCA distribution consistent with infarct MCA: middle cerebral artery

Radiograph of the chest was normal with no evidence of edema or cardiomegaly. Initial CT brain without contrast showed no abnormalities, however, a CT angiography of the head with contrast displayed a right middle cerebral artery (MCA) occlusion with penumbra of the right temporal and parietal lobes. Repeat CT brain without contrast revealed an evolving right MCA distribution infarct (Figure 2).

The patient underwent a mechanical thrombectomy for an M1 occlusion of the middle cerebral artery. Catheterization was performed with a femoral artery puncture. The catheter was guided to the occlusion located in the right MCA. The stent retriever was subsequently deployed and grabbed the clot. This removed the clot as the device was pulled back. There was complete recanalization of the middle cerebral artery with a subsequent thrombolysis in cerebral infarction (TICI) score of 3, indicating that there was complete reperfusion of the downstream target arterial territory, including the distal branches. In addition, there was a small stroke in the distal aspect of the anterior cerebral artery that was not targeted by thrombectomy. Subsequent brain MRI without contrast revealed a mild right frontal subarachnoid hemorrhage.

**Figure 3:**
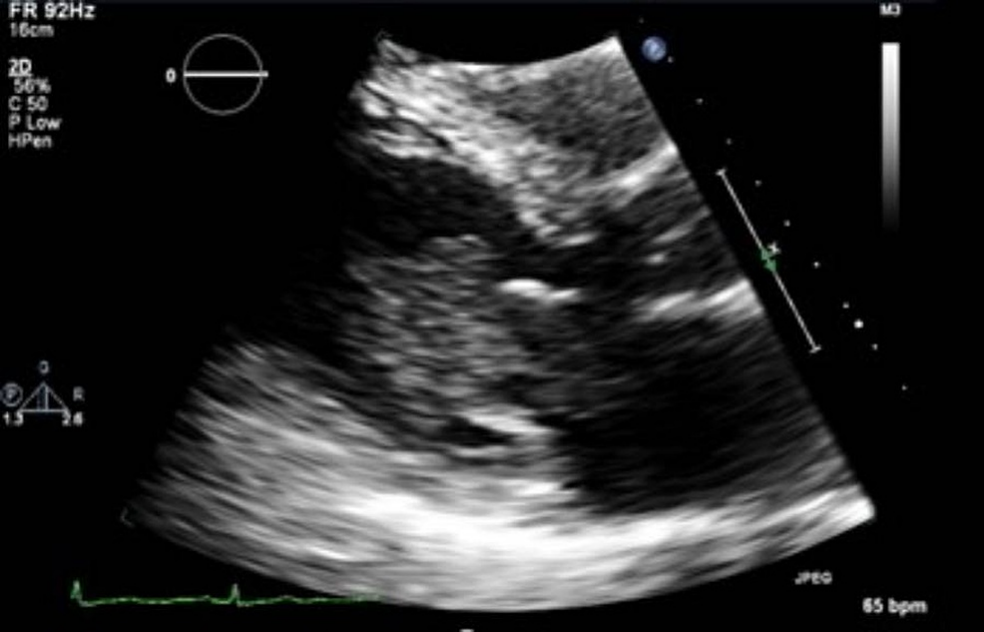
Echocardiogram showing ejection fraction >55%, enormous left atrial myxoma, and trace mitral regurgitation

Cardiac workup included an echocardiogram, which illustrated an enormous left atrial myxoma with normal left ventricle function (Figure 3).

**Figure 4:**
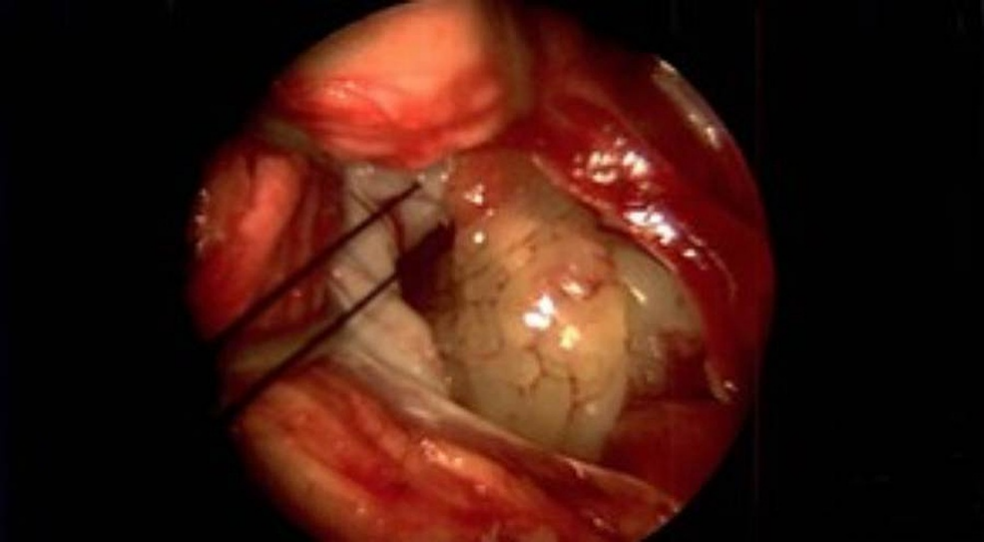
Left atrial myxoma during surgical resection

The myxoma was located around the fossa ovalis with an approximate 1.5 cm stalk. The myxoma was almost 8 cm in length and was transitioning from the left atrium into the left ventricle. Surgical intervention was successful in removing the myxoma in its entirety and in repairing a small atrial septal defect at the level of the fossa ovalis (Figure 4). Surgical Pathology utilized calretinin immunostains to highlight stellate myxoma cells. There was associated calcification, fibrosis, and fibrin present in the tumor.

Postoperatively, the patient was monitored closely in the ICU. She was noted to have hyperglycemia postoperatively where an insulin drip was started, and endocrinology was consulted. The patient’s hemoglobin A1c (HbA1c) was noted to be 5.3%. The patient’s blood glucose stabilized and did not require further intervention. She was stable and was transferred to the medical floor with telemetry monitoring on postoperative day 5. After her transfer, the patient’s chest x-ray showed perihilar opacities that were suspected to be pneumonitis. The patient was treated empirically with intravenous Rocephin and Flagyl. The patient also developed an episode of atrial fibrillation with a rapid ventricular response. Amiodarone drip was started and subsequently transitioned to oral administration twice daily. The patient did not have further episodes of atrial fibrillation with amiodarone. The patient continued to improve clinically. She was able to mobilize out of bed with physical therapy. The patient required minimal assistance for supine to sit, sit to stand, and moderate assistance to ambulate with a right hemi walker. Patient was discharged to an acute rehab center to continue her recovery.

## Discussion

Despite being extremely rare, cardiac myxomas are the most common primary cardiac tumor, with around 70% presenting in the LA [3, 11]. More than half of cardiac myxomas are benign in nature but can manifest with embolic or obstructive symptoms [3, 6, 9, 11].

Iyer et al. reported a similar case of a patient with left atrial myxoma presenting with ischemic stroke who was having symptoms of palpitations for a year prior to diagnosis [11]. Our patient was having symptoms of chest pain and shortness of breath with mild exertion for several weeks prior to her presentation with slurred speech and left-sided weakness.

Our patient’s diagnosis of left atrial myxoma was supported by the echocardiogram displaying an enormous tumor intermittently projecting through the mitral valve. Surgical resection of the myxoma revealed a remarkably sized tumor, measuring 8 cm, on a short stalk. The size of the mass is to be well-regarded as very large in comparison to other case reports. This unusual cause of acute ischemic stroke as seen in our patient accounts for less than 1% in the general population [12]. There have been studies illustrating that 27.7% of patients with cardiac myxomas had ischemic strokes directly related to the myxoma [13]. Patients are at a risk for recurrence of the tumor or additional lesions; therefore, patients are carefully followed up after recovery [5].

## Conclusions

We present this case to highlight the importance of investigating secondary causes of a cerebrovascular accident. Cardiovascular workup, including EKG and echocardiogram, should be included in the initial investigation. These tests are relatively inexpensive and simple to obtain. Transthoracic echocardiogram (TTE) is useful for initial diagnosis while a transesophageal echocardiogram (TEE) is beneficial for operative removal of the tumor. In this case, the pre-operative TEE was instrumental in identifying the 1.5 cm stalk near the fossa ovalis, allowing for quick surgical intervention. This case was unique in that the size of the myxoma was larger than any in the reported literature. With a size of 8 cm, this myxoma was still able to easily flow into the left ventricle with diastolic filling and then flow back into the atrium upon systole. This case highlights the importance of considering cardiac myxomas in patients presenting with stroke symptoms. Quick and effective cardiac workup should be performed so that clinicians can diagnose and surgically remove these rare primary tumors, in order to reduce morbidity and mortality.
